# Intracellular trafficking pathways for plasmid DNA complexed with highly efficient endosome escape polymers

**DOI:** 10.1186/1753-6561-9-S9-P69

**Published:** 2015-12-14

**Authors:** Marianne Gillard, Zhongfan Jia, Jeff Hou, Michael Song, Peter P Gray, Trent P Munro, Michael J Monteiro

**Affiliations:** 1 Australian Institute for Bioengineering and Nanotechnology, The University of Queensland, Brisbane QLD 4072, Australia; 2 Current address: Amgen Inc. One Amgen Center Drive, Thousand Oaks CA 91320 USA

## Background

Non-viral gene delivery vectors are widely used for the delivery of genetic materials into mammalian cells. Currently, there is a need to develop cheap and efficient transfection agents for use in production of recombinant proteins via transient gene expression. There are several barriers that non-viral vectors must overcome for successful transfection, these include cellular internalisation, endosome escape, protection of DNA and delivery of DNA into the nucleus. The ability to escape the endosome and gain entry to the nucleus are of the two primary barriers to successful transfection. The processes involved in the pathways for cellular uptake, intracellular trafficking, and nuclear entry are still not fully understood. More detailed understanding of the pathways involved in transfection is needed in order to develop highly efficient transfection agents.

## Materials and methods

**Figure 1:**
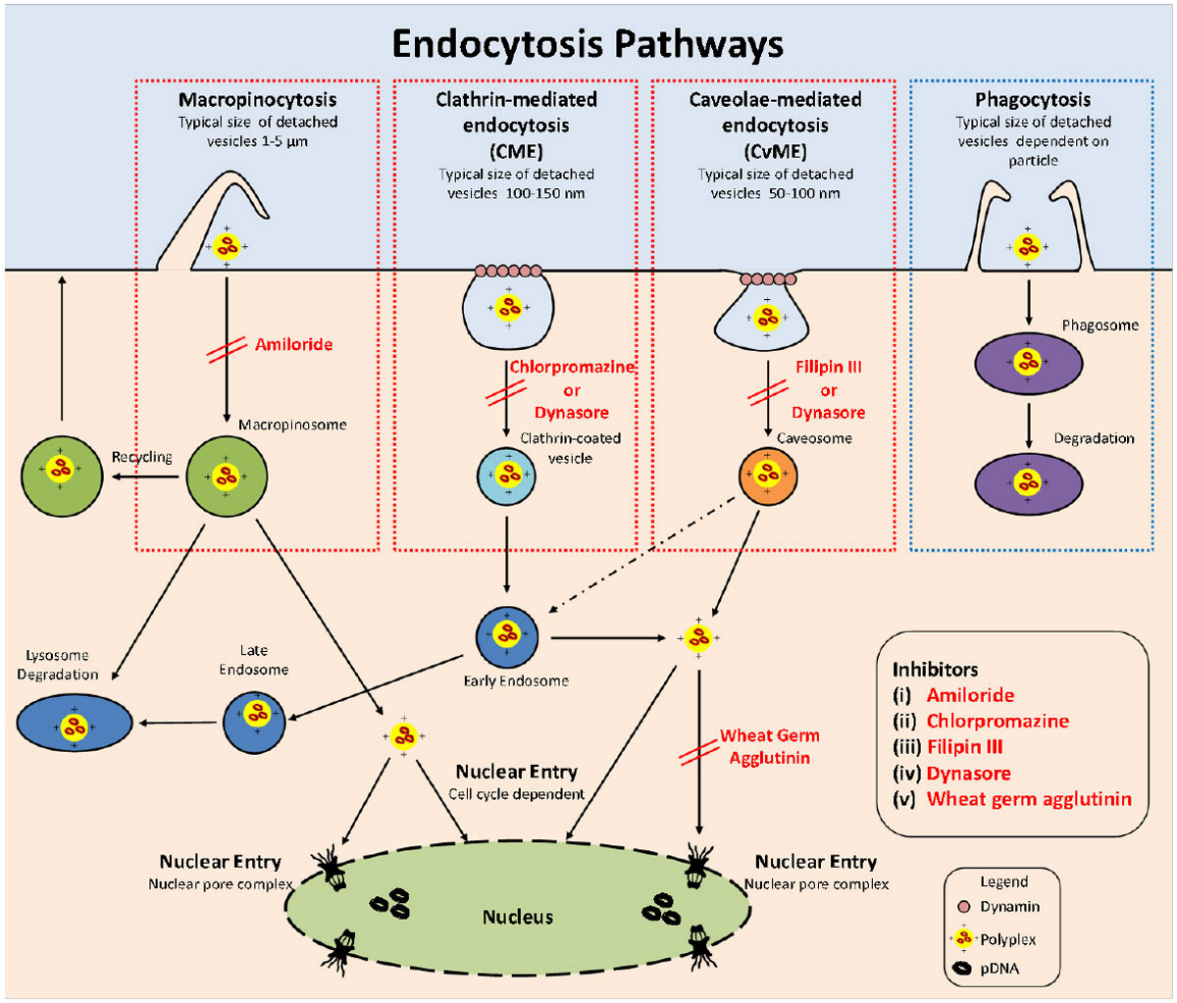
**Pathways involved in the cellular and nuclear uptake of polyplexes and lipoplexes, and inhibitors used to stop these pathways**.^1^

This work investigates the use of three series of cationic diblock copolymers as transfection agents for the production of recombinant proteins, as well as examining the pathways the polymers used for delivery of DNA into the nucleus. The diblock copolymers were synthesised using 'living' radical polymerization techniques, with each series using the same first block poly(2-dimethylaminoethyl acrylate) (PDMAEA). This polymer can self-degrade through a self-catalysed hydrolysis mechanism to a negatively charge and nontoxic poly(acrylic acid) in a time-dependent manner. The second block consists of N-(3-(1H-imidazol-1-yl)propyl) acrylamide (ImPAA) or butylacrylate (BA) or a combination of both. The three series of polymers were first tested for their ability to bind/release and protect pDNA before transfection studies occurred. Transfection studies were performed in Human Embryonic Kidney (HEK293) cells where internalisation pathways into the cell, endosomal escape and nuclear entry were investigated before recombinant protein yields determined. Several chemicals were used to investigate the internalisation pathways and endosomal escape (see Figure 1). Uptake of polymer/pDNA polyplexes were investigated though the use of specific inhibitors to block endocytosis pathways (chlorpromazine, filipin III, dynasore and amiloride). The ability of the polymer/pDNA complexes to escape the endosome was determined by the addition of chloroquine, a chemical known to swell and burst endosomes. And finally to investigate the nuclear entry pathway for pDNA, either complexed or alone, wheat germ agglutinin was used.

## Results

Polymer A-C3, with the second block copolymer of both the ImPAA and BA not only showed the best protection against DNase I with a timed-release mechanism between 24-48 h, but also achieved the highest level of transfection efficiency with 95% of HEK293 cells testing positive for gene expression. To understand the pathways involved in the delivery of pDNA within the cell and the nucleus, several different chemical inhibitors were employed. The addition of the endosome inhibitor, chlorpromazine, resulted in approximately 80% decrease in the number of cells testing positive for gene expression, indicating that the main internalisation pathway into the cell was clathrin-mediated endocytosis. The ability of the polymer/pDNA complexes to escape the endosome was tested through the addition of chloroquine, a chemical known to swell and burst endosomes releasing its contents into the cytosol. Our results show that the polymer A-C3 is efficient at endosomal escape as no increase in transfection efficiency was seen. The final pathway tested was nuclear entry. Nuclear entry of the pDNA is thought to occur either through the nuclear pores or during mitosis when the nuclear membrane is temporarily disintegrated. To determine how the pDNA enters the nucleus, wheat germ agglutinin (WGA), which is known to block the nuclear pores, was added before transfection. Our results show that 48 h post-transfection, transfection efficiency is significantly reduced to less than 5%. We confirmed that reduced transfection efficiency is not due to any toxic effects caused by the WGA. Our results demonstrate that entry occurs primarily though the nuclear pores, and not during mitosis when the nuclear membrane is temporarily disintegrated as the cells would have undergone at least one cell division during the 48 h period.

## Conclusions

The results presented here attempts to improve our understanding of the pathways involved in the successful delivery of pDNA. The three series of cationic diblock copolymers investigated are highly effective at escaping the endosome with polymer A-C3 achieving the highest level of transfection efficiency. The A-C3 polymer/pDNA complexes showed a preference to the clathrin-mediated endocytosis (CME) cellular entry pathway. Through the addition of WGA it can be suggested that pDNA either complexed with the A-C3 polymer or alone, enters the nucleus via the nuclear pores and not during mitosis. The ability to rationally design cationic polymers to overcome the barriers to successful transfection could result in the next generation of highly efficient transfection agents used in transient gene expression systems.
